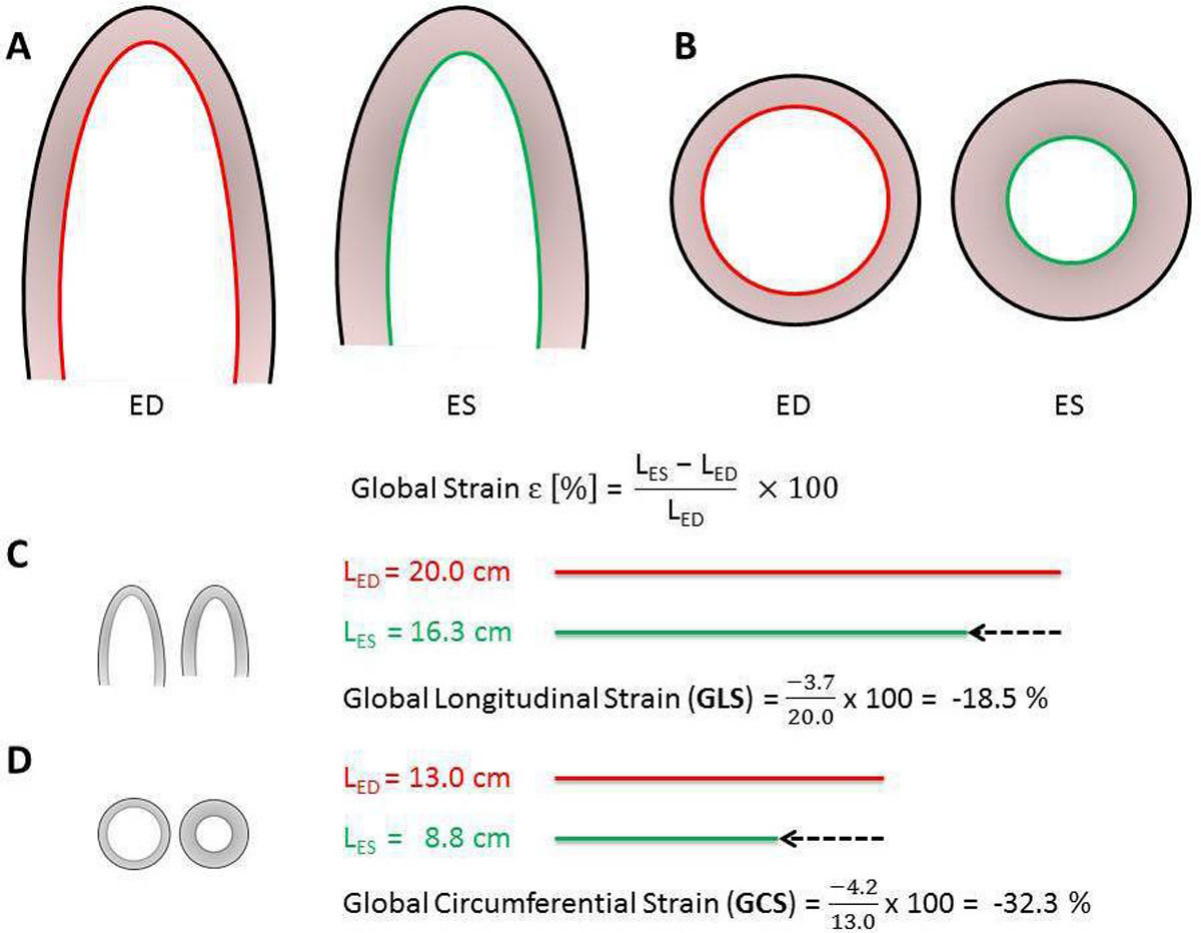# Standardized assessment of global longitudinal and circumferential strain - a modality independent software approach

**DOI:** 10.1186/1532-429X-17-S1-Q9

**Published:** 2015-02-03

**Authors:** Johannes Riffel, Marius G Keller, Matthias Aurich, Yannick Sander, Florian Andre, Sorin Giusca, Fabian aus dem Siepen, Sebastian Seitz, Christian Galuschky, Grigorios Korosoglou, Derliz Mereles, Hugo Katus, Sebastian Buss

**Affiliations:** 1 Cardiology, University of Heidelberg, Heidelberg, Germany; 2 TomTec, Unterschleissheim, Germany

## Background

Myocardial deformation measurement is superior to left ventricular ejection fraction in identifying early changes in myocardial contractility and prediction of cardiovascular outcome. The lack of standardization hinders its clinical implementation.

The aim of the study is to investigate a novel standardized deformation imaging approach based on the feature tracking algorithm for the assessment of global longitudinal (GLS) and global circumferential strain (GCS) in echocardiography and cardiac magnetic resonance imaging (CMR).

## Methods

70 subjects undergoing CMR were consecutively investigated with echocardiography within a median time of 30 min. GLS and GCS were analyzed with a post processing software incorporating the same standardized algorithm for both modalities. Accordingly, global strain was defined as the relative shortening of the whole, uni-segmented endocardial contour and calculated according to the strain formula.

## Results

Mean GLS values were -16.2±5.3% and -17.3±5.3% for echocardiography and CMR, respectively. GLS did not differ significantly between the two imaging modalities, which showed strong correlation (r=0.86), a small bias (-1.1%) and narrow 95% limits of agreement (LOA, ±5.4%). Mean GCS values were -17.9±6.3% and -24.4±7.8% for echocardiography and CMR, respectively. GCS was significantly underestimated by echocardiography (p<0.001). A weaker correlation (r=0.73), a higher bias (-6.5%) and wider LOA (±10.5%) were observed for GCS. GLS showed a strong correlation (r=0.92) when image quality was good, while correlation dropped to r=0.82 with poor acoustic windows in echocardiography. GCS assessment revealed only a strong correlation (r=0.87) when echocardiographic image quality was good. No significant differences for GLS between two different echocardiographic vendors could be detected.

## Conclusions

Quantitative assessment of GLS using a standardized software algorithm allows the direct comparison of values acquired irrespective of the imaging modality. GLS may therefore serve as a reliable parameter for the assessment of global left ventricular function in clinical routine besides standard evaluation of the ejection fraction.

## Funding

The study was supported by a grant from the B. Braun Stiftung. H.A.K. was supported by the DZHK (Deutsches Zentrum für Herz-Kreislauf-Forschung - German Centre for Cardiovascular Research).

**Figure 1:**